# Diabetic Foot Complications and Their Risk Factors from a Large Retrospective Cohort Study

**DOI:** 10.1371/journal.pone.0124446

**Published:** 2015-05-06

**Authors:** Khalid Al-Rubeaan, Mohammad Al Derwish, Samir Ouizi, Amira M. Youssef, Shazia N. Subhani, Heba M. Ibrahim, Bader N. Alamri

**Affiliations:** 1 University Diabetes Center, College of Medicine, King Saud University, Riyadh, Saudi Arabia; 2 Diabetic Foot Unit, University Diabetes Center, King Saud University, Riyadh, Saudi Arabia; 3 Registry Department, University Diabetes Center, King Saud University, Riyadh, Saudi Arabia; 4 Department of Biostatistics, Epidemiology and Scientific Computing, King Faisal Specialist Hospital and Research Centre, Riyadh, Saudi Arabia; 5 Internal Medicine Department, Dalhousie University, Halifax, Nova Scotia, Canada; Sapienza, University of Rome, School of Medicine and Psycology, ITALY

## Abstract

**Background:**

Foot complications are considered to be a serious consequence of diabetes mellitus, posing a major medical and economical threat. Identifying the extent of this problem and its risk factors will enable health providers to set up better prevention programs. Saudi National Diabetes Registry (SNDR), being a large database source, would be the best tool to evaluate this problem.

**Methods:**

This is a cross-sectional study of a cohort of 62,681 patients aged ≥25 years from SNDR database, selected for studying foot complications associated with diabetes and related risk factors.

**Results:**

The overall prevalence of diabetic foot complications was 3.3% with 95% confidence interval (95% CI) of (3.16%–3.44%), whilst the prevalences of foot ulcer, gangrene, and amputations were 2.05% (1.94%–2.16%), 0.19% (0.16%–0.22%), and 1.06% (0.98%–1.14%), respectively. The prevalence of foot complications increased with age and diabetes duration predominantly amongst the male patients. Diabetic foot is more commonly seen among type 2 patients, although it is more prevalent among type 1 diabetic patients. The Univariate analysis showed Charcot joints, peripheral vascular disease (PVD), neuropathy, diabetes duration ≥10 years, insulin use, retinopathy, nephropathy, age ≥45 years, cerebral vascular disease (CVD), poor glycemic control, coronary artery disease (CAD), male gender, smoking, and hypertension to be significant risk factors with odds ratio and 95% CI at 42.53 (18.16–99.62), 14.47 (8.99–23.31), 12.06 (10.54–13.80), 7.22 (6.10–8.55), 4.69 (4.28–5.14), 4.45 (4.05–4.89), 2.88 (2.43–3.40), 2.81 (2.31–3.43), 2.24 (1.98–2.45), 2.02 (1.84–2.22), 1.54 (1.29–1.83), and 1.51 (1.38–1.65), respectively.

**Conclusions:**

Risk factors for diabetic foot complications are highly prevalent; they have put these complications at a higher rate and warrant primary and secondary prevention programs to minimize morbidity and mortality in addition to economic impact of the complications. Other measurements, such as decompression of lower extremity nerves, should be considered among diabetic patients.

## Introduction

Diabetic foot complications are contributing to both mortality and morbidity among the diabetic population leading to substantial physical, physiological and financial burden for the patients and community at large. It is estimated that 24.4% of the total health care expenditure among diabetic population is related to foot complications [1] and the total cost of treating diabetic foot complications is approaching 11 billion USD in USA [2] and 456 million USD in UK [3].

The risk of ulceration and amputation among diabetic patients increases by two to four folds with the progression of age and duration of diabetes regardless of the type of diabetes [4]. It has also been proven by many longitudinal epidemiological studies that among diabetic patients, the life time foot ulcer risk is about 25%[5,6], thereby accounting for two thirds of all non-traumatic amputations [7].

Foot ulceration is a preventable condition, where simple interventions can reduce amputations by up to 70%through programs that could reduce its risk factors [8]. Identifying the role of risk factors contributing to this condition will enable health providers to set up better prevention programs that could result in improving patients' quality of life and henceforth, reducing the economic burden for both the patient and the health care system.

Disease registries are currently considered to be a reliable source to monitor chronic diseases, such as diabetes, and their complications. Countries like Denmark, Sweden, Singapore, Malaysia, Saudi Arabia, and Thailand have adopted diabetes registries to monitor this disease [9–14].In this study, the Saudi National Diabetes Registry (SNDR), being one of the largest diabetes registries, was used to study the prevalence of foot ulcer, gangrene, and amputation and their risk factors among Saudi type 1 and type 2 diabetic patients aged 25 years and above.

## Material and Methods

### Study population

SNDR is a specially designed electronic web-based data system which incorporates demographic data and diabetes related clinical and biochemical parameters. The design and development of the web-based SNDR has already been explained in a previously published paper [15].

**Fig 1:**
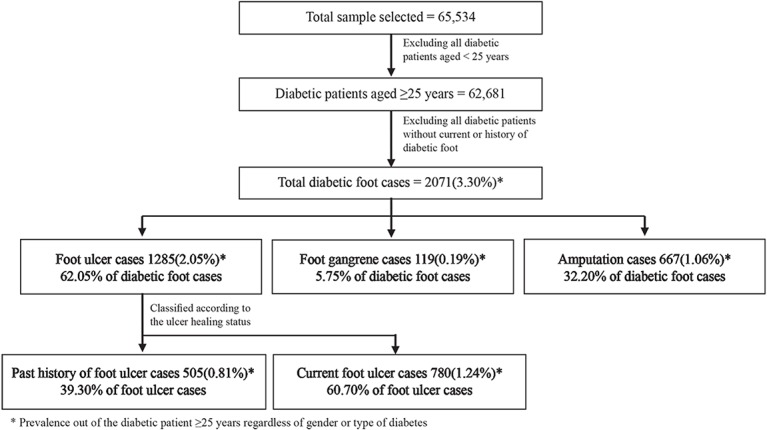
Classification of diabetic patients sample from Saudi National Diabetes Registry (SNDR) aged ≥25 years according to diabetic foot status.

A cross-sectional sample of anonymous 65,534 Saudi diabetic patients was selected from the start of SNDR in 2000 till December 2012. In this observational hospital-based study, a cohort of 62,681 diabetic patients aged ≥25 years were selected to study foot complications and related risk factors. A total of 2,071(3.3%) diabetic patients were found to have current or history of diabetic foot ulcer, gangrene or diabetes related lower limb amputation as shown in Fig 1.

### Case identification

Data were collected from patients' hospital charts including demographic, social and anthropometric data. Diabetes mellitus related data including type, duration and the most recent management i.e. oral hypoglycemic agents, insulin, or both, were also collected. Diabetes glycemic parameters namely; HbA1c, fasting blood sugar (FBG) and random blood sugar (RBS) were collected from patients' laboratory data according to their latest hospital visit. Any associated diseases including hypertension and hyperlipidemia were also reported.

Chronic complications namely vasculopathy including PVD, CVD and CAD, retinopathy, nephropathy, and neuropathy were reported for their presence. PVD was defined based on either clinical and physical examination documented in patient's file, i.e. absent or diminished pulses, abnormal skin color, poor hair growth, and cool skin. or through ABI measurements, where ABI value of 0.70–0.90 was considered as mild occlusion and ABI value of <40 as a sever occlusion. CVD; was defined based on the neurological assessment documented in the patients’ files, CVD was considered if clinical symptoms indicated a rapid developing neurological deficit that persisted for more than 24 hours, or led to death in the absence of other conditions that could explain the symptoms. CAD was defined based on the history of hospital admission for either myocardial infarction (MI) or angina, positive ECG for prior MI or angina, and positive history of coronary artery bypass grafting or percutaneous transluminal coronary angioplasty. Retinopathy was defined as non-proliferative diabetic retinopathy (NPDR) and proliferative diabetic retinopathy (PDR) according to the clinical diagnosis documented in patients’ file, while the level of retinopathy was based on the grading of the worst eye. Nephropathy was defined by the albumin excretion in urine as microalbuminuria when albumin was between 30–299 μg\mg creatinine, and macroalbuminuria when albumin excretion was≥300 μg\mg creatinine. Patients were identified with ESRD if they had GFR <30 ml\min per 1.73 m^2^ body surface area. Neuropathy was considered when the patients was suffering from any forms of diabetic neuropathy mainly diabetic polyneuropathy presented by numbness or pain or through clinical examination using monofilament, vibration and position and temperature sensation represented by current or past history of foot ulcer, gangrene or amputation. Foot ulcer was considered in diabetic patients with current or history of non-healing or poorly healing partial or full skin thickness wound below the ankle. Foot gangrene was diagnosed when there was tissue death and decay, as a result of ischemia related to the foot, proven by Doppler study. Charcot joint was considered when bones, joints, and soft tissues of the foot and ankle are inflamed in the presence of neuropathy with or without history of trauma leading to variable degrees of bone destruction, subluxation, dislocation, and deformity [16]. Amputation was reported, if the patient had a minor distal or a major proximal amputation that was related to diabetes [17].

SNDR is one of the strategic research projects of Saudi Arabia that was funded by King Abdulaziz City for Science and Technology (KACST) and approved by KACST institutional review board. SNDR can be accessed at http://www.diabetes.org.sa. This web application, however, is available for authorized users only. The data used in this publication was not consented since it does not compromise anonymity or confidentiality or breach local data protection laws. In addition, the patients' records / information were anonymized and de-identified prior to analysis.

### Statistical analysis

The study was designed and reported in accordance with Strengthening the Reporting of Observational Studies in Epidemiology (STROBE) guidelines. All data were analyzed using SPSS program version 17.0. Chi square test (χ2) was used for categorical variables such as gender and smoking status, while t-test was used for continuous variables. A p-value of 0.05 or less was used as a level of significance. Odds ratio(OR) with their 95% confidence interval (CI)were used as for assessing the risk factors of diabetic foot complications using the univariate analysis, while age and gender adjusted in addition to multivariate logistic regression analysis were used to control for any potential confounders.

## Results

### Prevalence

The overall prevalence of diabetic foot complications among the diabetic patients cohort was found to be at 3.3%wherein, it was distributed as 2.05%, 0.19%, and 1.06% for foot ulcer, gangrene, and amputation respectively regardless of their gender or type of diabetes (Fig 1). Out of the total of 2071 registered diabetic foot cases,1285 (62.05%) were foot ulcer cases divided into 39.30% cases with past history of ulcer and 60.70% cases with current ulcer, while 119 (5.75%) and 667 (32.20%) were foot gangrene and amputation cases.

### Demographic characteristics

Diabetic foot ulcer, gangrene, and amputation cases were significantly older than the non affected diabetic patients at 62.97±12.70, 63.66±12.52, and 65.35±12.37 years respectively, and had significantly lower BMI at 29.23±6.26, 28.77±7.38, and 29.47±6.19 kg/m^2^respectively. The duration of diabetes was significantly higher in foot ulcer, gangrene, and amputation cases when compared with the non-affected patients. HbA1c was also found to be significantly higher in all the three different types of diabetic foot complications when compared with non-affected patients, and the results were also the same for FBS and RBS. See Table 1.

**Table 1:** Mean (SD) for clinical and biochemical characteristics of the selected cohort aged ≥ 25 years according to the diabetic foot state.

Characteristics	Total sample Mean±SD	Total non-affected Mean±SD	Total affected Mean±SD	^a^P value	Diabetic Foot							
					Foot ulcer			^a^P value	Gangrene Mean±SD	^a^P value	Amputation Mean±SD	P value
					Current Mean±SD	Past history Mean±SD	Total Mean±SD					
Number	62,681	60610	2071		780	505	1285		119		667	
Age (years)	56.91±13.54	56.68±13.51	63.78±12.63	<0.0001	63.72±12.48	61.82±12.97	62.97±12.70	<0.0001	63.66±12.52	< 0.0001	65.35±12.37	< 0.0001
Weight (kg)	78.86±16.61	78.91±16.60	77.08±16.80	<0.0001	76.45±16.41	78.58±15.96	77.10±16.29	0.001	76.11±19.28	0.257	77.18±17.50	0.032
Height (cm)	160.11±9.58	160.05±9.57	162.07±9.51	<0.0001	162.28±9.18	161.91±10.08	162.17±9.45	<0.0001	162.63±9.19	0.042	161.76±9.70	0.001
BMI (kg/m^2^)	30.63±6.40	30.67±6.40	29.28±6.28	<0.0001	29.03±6.38	29.71±5.91	29.23±6.26	<0.0001	28.77±7.38	0.034	29.47±6.19	< 0.0001
DM duration (years)	13.29±8.10	13.02±7.98	20.53±7.96	<0.0001	20.61±8.16	19.17±7.41	20.07±7.91	<0.0001	19.47±8.37	< 0.0001	21.58±7.91	< 0.0001
HbA1c (%)	8.82±2.37	8.80±2.36	9.91±2.33	<0.0001	10.12±2.22	9.52±2.06	9.91±2.18	<0.0001	10.80±2.53	< 0.0001	9.66±2.72	0.001
FBS (mmol/L)	9.95±4.28	9.91±4.25	11.46±5.08	<0.0001	11.31±4.8	11.79±5.34	11.50±5.04	<0.0001	11.15±5.23	< 0.0001	11.39±5.17	< 0.0001
RBS (mmol/L)	12.65±5.40	12.62±5.39	13.38±5.51	<0.0001	13.66±5.33	13.04±5.66	13.41±5.47	0.003	14.03±5.32	< 0.0001	13.19±5.65	<0.0001

^a^P value was calculated using non-diabetic foot cohort

As shown in Table 2, the percentages of the affected cases were found to be 7.20%, 40.80% and 52.00% for age groups 25–44, 45–64, and ≥65 years respectively. Foot ulcer and gangrene percentages did not differ much between the age groups, except for amputation which had shown increased percentage among older age groups. The frequency of different diabetic foot complications was found to be similar for both the genders, although males were affected more than females presented by 68.57% and 31.43%. The majority of cases were married accounting for 91.45% and family history of diabetes was 40.42% for the total affected cases which is similar to non-affected patients and the total sample. Smoking was significantly higher among diabetic foot cases at 10.14% versus 6.72% for non-affected cases, and the percentage of smokers was significantly higher among foot ulcer and gangrene cases, but not among amputees. There were more affected diabetic foot cases with higher BMI at 23.23%, 36.02%, and 40.75% in BMI groups <25, 25–29.9, ≥ 30 kg/m^2^ respectively, and more obesity was observed among ulcer cases when compared with gangrene and amputation. There were more type 2 diabetic patients among total diabetic foot cases (94.27%), who also had more gangrene and amputation cases. Out of the total diabetic foot cases, 88.99% had diabetes duration of more than 10 years. The number of cases for foot ulcer, gangrene, and amputation increased with longer duration, although the percentage between each duration group decreased for ulcer and gangrene, but increased among the amputation cases.

**Table 2:** Number and percentage of different clinical and biochemical characteristics of the selected patients aged ≥ 25 years according to the diabetic foot state.

Characteristics		Total sample n(%)	Non-affected n(%)	Affected n(%)	Diabetic Foot							
					Foot ulcer			^a^P value	Gangrene n(%)	^a^P value	Amputation n(%)	^a^P value
					Current n(%)	Past history n(%)	Total n(%)					
Number		62,681	60610	2071	780	505	1285		119		667	
Age:	25–44 years	11,197(17.9)	11,048(18.23)	149(7.20)	53(35.57)	53(35.57)	106(71.14)	<0.0001	8(5.37)	0.007	35(23.49)	<0.0001
	45–64 years	31,914(50.9)	31,069(51.26)	845(40.80)	317(37.51)	225(26.63)	542(64.14)	<0.0001	48(5.68)	0.017	255(30.18)	<0.0001
	≥65 years	19,570(31.2)	18,493(30.51)	1,077(52.00)	410(38.07)	227(21.08)	637(59.15)	<0.0001	63(5.85)	<0.0001	377(35.00)	<0.0001
Gender:	Male	32,868(52.4)	31,448(51.89)	1,420(68.57)	553(38.94)	324(22.82)	877(61.76)	<0.0001	78(5.49)	0.003	465(32.75)	<0.0001
	Female	29,813(47.6)	29,162(48.11)	651(31.43)	227(34.87)	181(27.80)	408(62.67)	<0.0001	41(6.30)	0.003	202(31.03)	<0.0001
Marital Status:	Single	2,150(3.43)	2,096(3.46)	54(2.61)	23(42.59)	16(29.63)	39(72.22)	<0.0001	0.0(0.00)	0.038	15(27.78)	0.089
	Married	57,325(91.46)	55,431(91.45)	1,894(91.45)	704(37.17)	463(24.45)	1167(61.62)	0.418	116(6.12)	0.013	611(32.26)	0.891
	Divorced	641(1.02)	622(1.03)	19(0.92)	12(63.16)	3(15.79)	15(78.95)	0.620	1(5.26)	0.841	3(15.79)	0.173
	Widow	2,565(4.09)	2,461(4.06)	104(5.02)	41(39.42)	23(22.12)	64(61.54)	0.099	2(1.92)	0.189	38(36.54)	0.034
Family history of DM	Yes	25,187(40.18)	24,350(40.17)	837(40.42)	365(43.61)	167(19.95)	532(63.56)	0.010	44(5.26)	0.554	261(31.18)	0.671
Smoking	Yes	4,286(6.84)	4,076(6.72)	210(10.14)	77(36.67)	65(30.95)	142(67.62)	<0.0001	15(7.14)	0.039	53(25.24)	0.260
BMI	<25 kg/m^2^	10,743(17.14)	10,262(16.93)	481(23.23)	208(43.24)	100(20.79)	308(64.03)	<0.0001	40(8.32)	<0.0001	133(27.65)	0.038
	25–29.9 kg/m^2^	20,855(33.27)	20,109(33.18)	746(36.02)	264(35.39)	179(23.99)	443(59.38)	0.329	37(4.96)	0.629	266(35.66)	<0.0001
	≥30 kg/m^2^	31,083(49.59)	30,239(49.89)	844(40.75)	308(36.49)	226(26.78)	534(63.27)	<0.0001	42(4.98)	<0.0001	268(31.75)	<0.0001
Diabetes type	Type 1	2,604(4.55)	2,486(4.50)	118(5.73)	50(42.37)	37(31.36)	87(73.73)	<0.0001	1(0.85)	<0.0001	30(25.42)	0.002
	Type 2	54,669(95.45)	52,727(95.50)	1,942(94.27)	726(37.38)	464(23.89)	1190(61.28)	<0.0001	118(6.08)	<0.0001	634(32.65)	<0.0001
Diabetes duration	<5 years	8,101(12.92)	8,062(13.30)	39(1.88)	18(46.15)	8(20.51)	26(66.67)	<0.0001	5(12.82)	0.003	8(20.51)	<0.0001
	5–10 years	18,844(30.06)	18,655(30.78)	189(9.13)	68(35.98)	60(31.75)	128(67.72)	<0.0001	16(8.47)	<0.0001	45(23.81)	<0.0001
	>10 years	35,736(57.01)	33,893(55.92)	1,843(88.99)	694(37.66)	437(23.71)	1131(61.37)	<0.0001	98(5.32)	<0.0001	614(33.32)	<0.0001
Diabetes chronic complications	Neuropathy	11,153(17.79)	9,096(15.01)	2,057(99.32)	776(37.72)	499(24.26)	1275(61.98)	<0.0001	119(5.79)	<0.0001	663(32.23)	<0.0001
	Retinopathy	11,262(17,97)	10,296(16.99)	966(46.64)	385(39.86)	181(18.74)	566(58.59)	<0.0001	37(3.83)	<0.0001	363(37.58)	<0.0001
	Nephropathy	6,252(9.97)	5,644(9.31)	608(29.36)	250(41.12)	91(14.97)	341(56.09)	<0.0001	25(4.11)	<0.0001	242(39.80)	<0.0001
	Vascylopathy	10,384(16.57)	9,698(16.00)	686(33.12)	234(34.11)	146(21.28)	380(55.39)	<0.0001	52(7.58)	<0.0001	254(37.03)	<0.0001
Vasculopathy	CAD	8,020(12.79)	7,532(12.43)	488(23.56)	154(31.56)	106(21.72)	260(53.28)	<0.0001	32(6.56)	<0.0001	196(40.16)	<0.0001
	CVD	2,769(4.42)	2,558(4.22)	211(10.19)	55(26.07)	41(19.43)	96(45.50)	<0.0001	27(12.80)	<0.0001	88(41.71)	<0.0001
	PVD	197(0.31)	147(0.24)	50(2.41)	9(18.00)	6(12.00)	15(30.00)	<0.0001	8(16.00)	<0.0001	27(54.00)	<0.0001
Hypertension	Yes	29,399(46.90)	2,8223(46.56)	1,176(56.78)	435(36.99)	253(21.51)	688(58.50)	<0.0001	78(6.63)	<0.0001	410(34.86)	<0.0001
Hyperlipidemia	Yes	22,701(36.22)	22,060(36.40)	641(30.95)	266(41.50)	157(24.49)	423(65.99)	0.012	37(5.77)	0.256	181(28.24)	<0.0001
Treatment	Oral agent only	39,600(63.18)	39,020(64.38)	580(28.01)	222(38.27)	169(29.14)	391(67.41)	<0.0001	33(5.69)	<0.0001	156(26.90)	<0.0001
	Insulin only	15,691(25.03)	14,559(24.02)	1,132(54.66)	404(35.69)	247(21.82)	651(57.51)	<0.0001	68(6.01)	<0.0001	413(36.48)	<0.0001
	Oral agent & insulin	7,390(11.79)	7,031(11.60)	359(17.33)	154(42.90)	89(24.79)	243(67.69)	<0.0001	18(5.01)	0.230	98(27.30)	0.013

^a^P value was calculated using non-diabetic foot cohort

Retinopathy and nephropathy were also more prevalent among diabetic foot cases than non-affected at 46.64% and 29.36% versus 16.99% and 9.31% respectively. Total vasculopathic cases were 33.12% among diabetic foot cases versus only 16% in non-affected cases. Among diabetic foot cases, CAD was the most prevalent type of vasculopathy at 23.56% followed by CVD and PVD at 10.19% and 2.41% respectively. However, PVD prevalence was high among different types of diabetic foot cases at a rate of 30% among total foot ulcer cases and 54% among amputation cases.

Hypertension affected 56.78% of the diabetic foot cases versus 46.56% for non- affected cases, which was not the case for hyperlipidemia, where more percentage of hypelipidemic patients were found in non-affected cases at 36.40% versus 30.95% for affected cases. There were more percentages of hypertensive patients in gangrene and amputation foot cases than ulcer ones. The percentage of oral hypoglycemic agents' users was higher among non-affected cases when compared with diabetic foot cases who were frequent insulin users. This was also the same observation among gangrene and amputation cases, but not for foot ulcer cases who were more on oral agents and insulin combination therapy

**Fig 2:**
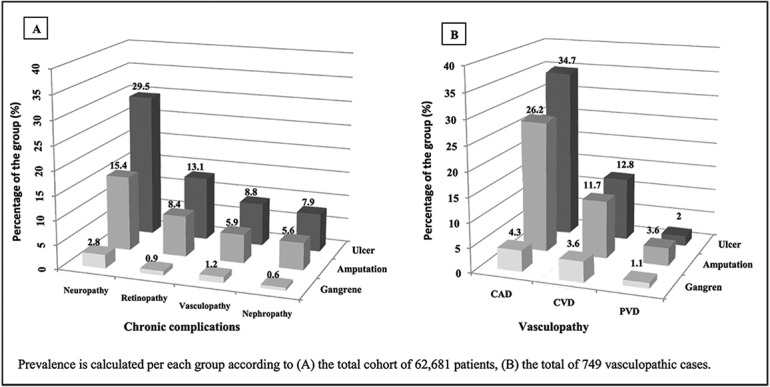
Effect of different diabetes chronic complications and types of vasculopathy on foot ulcer, gangrene, and amputation prevalence.

Neuropathy is the most frequent chronic complication in foot ulcer, amputation and gangrene cases followed by retinopathy, vasculopathy, and nephropathy respectively as shown in Fig 2A. At the same time, all these chronic complications were more frequent in foot ulcer cases followed by amputation and gangrene. When analyzing all cases with vasculopathy totaling to 749 cases, the same observation was noted, wherein foot ulcer cases had the highest percentage of CAD, CVD, and PVD followed by amputation and then gangrene cases, except for the percentage of PVD with amputation cases as shown in Fig 2B.

### Age and gender specific prevalence

**Fig 3:**
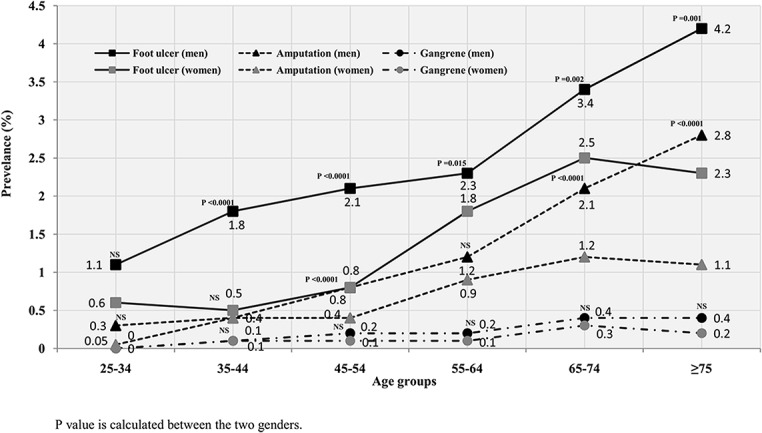
Age specific prevalence of diabetic foot disorders by gender for the total studied cohort of 62,681 diabetic patients.

Fig 3 demonstrates age specific prevalence of diabetic foot ulcer, amputation and gangrene according to gender, where foot ulcer prevalence increased with age, peaking at 4.2% for males ≥75 years of age and 2.5% for females in the age group 65 to 74 years. Foot ulcer prevalence was significantly higher among males in all age groups except for the age group from 25 to 34 years. The prevalence of foot amputation was also observed to be increasing with age but at a lower rate, being more among males peaking at 2.8% for males at ≥75 years of age and 1.2% for females aged 65 to 74 years. Among amputation cases, prevalence was significantly higher in males than females in the age group ≥65 years and the age group 45–54 years. Gangrene was the lowest in prevalence in all age groups, where it had started to increase after the age of ≥65 years with no significant difference between the two genders in all age groups.

### Risk factors

The presence of Charcot joint was the most important and significant risk factor when all types of diabetic foot conditions were included with OR (95% CI) at 42.53 (18.16–99.62), which was also true for both foot ulcer and amputation at 52.81 (21.42–130.19) and 30.42 (8.22–112.62). PVD was the second important risk factor for all affected, gangrene and amputation cases with OR (95% CI) at 14.47 (8.99–23.31), 62.07 (24.17–59.40), and 2.14 (10.24–39.61) respectively. Peripheral neuropathy was the second significant risk factor for foot ulcer with OR (95% CI) of 15.61 (13.41–18.18), but the third for all affected and gangrene cases at 12.06 (10.54–13.80) and 6.55 (3.51–12.22). Duration of Diabetes ≥10 years was a significant risk factor for all affected, foot ulcer, and gangrene cases, with higher OR in amputation cases at 9.74 (6.99–13.59). CVD had a significant OR for all affected foot ulcer, and amputation cases, with higher OR in gangrene cases at 7.62 (4.34–13.36). Insulin use, presence of nephropathy, and age ≥45 years have demonstrated a significant increased risk with OR more than 2 in all affected, foot ulcer, gangrene, and amputation cases. Other significant risk factors were poor glycemic control, CAD, male gender, smoking, and hypertension that had shown a significant p value except for smoking in the amputation cases. Hyperlipidemia, overweight, and obesity were associated with significantly decreased risk for all affected, ulcer, gangrene, and amputation cases, except for hyperlipidemia in gangrene cases and overweight among amputees. See Table 3.

**Table 3:** Univariate Odds ratio and confidence interval (95%CI) for all diabetic foot risk factors among studied cohort.

Risk factors	All diabetic foot		Foot ulcer		Gangrene		Amputation	
	Odds Ratio	^a^pvalue	Odds Ratio	^a^pvalue	Odds Ratio	^a^p-value	Odds Ratio	^a^pvalue
Charcot joint	42.53(18.16–99.62)	<0.0001	52.81(21.42–130.187)	<0.0001	-	-	30.42(8.22–112.62)	<0.0001
Peripheral vascular disease	14.47(8.99–23.31)	<0.0001	8.33(4.12–16.83)	<0.0001	62.07(24.17–159.40)	<0.0001	20.14(10.24–39.61)	<0.0001
Peripheral Neuropathy	12.06(10.54–13.80)	<0.0001	15.61(13.41–18.18)	<0.0001	6.55(3.51–12.22)	<0.0001	6.94(5.33–9.04)	<0.0001
DM Duration ≥10 yrs	7.22(6.10–8.55)	<0.0001	6.70(5.44–8.25)	<0.0001	4.00(2.23–7.18)	<0.0001	9.74(6.99–13.59)	<0.0001
Insulin use	4.69(4.28–5.14)	<0.0001	4.28(3.81–4.79)	<0.0001	4.36(3.00–6.33)	<0.0001	5.73(4.85–6.75)	<0.0001
Retinopathy	4.45(4.05–4.89)	<0.0001	3.93(3.49–4.43)	<0.0001	2.24(1.47–3.40)	<0.0001	6.24(5.31–7.32)	<0.0001
Nephropathy	4.05(3.66–4.47)	<0.0001	3.51(3.09–3.99)	<0.0001	2.59(1.67–4.03)	<0.0001	5.55(4.73–6.52)	<0.0001
Age ≥ 45 yrs	2.88(2.43–3.40)	<0.0001	2.48(2.03–3.03)	<0.0001	3.09(1.51–6.34)	<0.0001	4.03(2.86–5.66)	<0.0001
Cerebral vascular disease	2.81(2.31–3.43)	<0.0001	2.08(1.58–2.75)	<0.0001	7.62(4.34–13.36)	<0.0001	3.62(2.64–4.97)	<0.0001
Poor glycemic control	2.72(2.13–3.48)	<0.0001	3.35(2.47–4.54)	<0.0001	3.37(1.14–9.96)	0.029	1.44(0.91–2.30)	<0.0001
Coronary heart disease	2.24(1.98–2.54)	<0.0001	1.93(1.64–2.27)	<0.0001	2.83(1.72–4.67)	<0.0001	2.83(2.31–3.46)	<0.0001
Male gender	2.02(1.84–2.22)	<0.0001	1.99(1.77–2.24)	<0.0001	1.76(1.21–2.58)	0.003	2.14(1.81–2.52)	<0.0001
Smoking	1.54(1.29–1.83)	<0.0001	1.69(1.37–2.09)	<0.0001	1.98(1.02–3.85)	0.039	1.20(0.87–1.66)	0.260
Hypertension	1.51(1.38–1.65)	<0.0001	1.32(1.18–1.48)	<0.0001	2.16(1.48–3.15)	<0.0001	1.83(1.57–2.15)	<0.0001
Overweight	0.77(0.66–0.90)	0.001	0.71(0.59–0.85)	<0.0001	0.48(0.24–0.95)	0.032	1.02(0.77–1.36)	0.889
Obesity	0.58(0.50–0.67)	<0.0001	0.56(0.47–0.67)	<0.0001	0.36(0.19–0.70)	0.002	0.68(0.51–0.91)	0.009

^a^Risk assessed by univariate logistic regression analysis

In logistic regression model adjusted for age and gender for the whole studied cohort, these risk factors were found in the same order as in the univariate analysis with a significant OR, except for smoking which showed a non-significant OR (95% CI) of 1.15 (0.96–1.37). When performing a multivariate logistic regression analysis, peripheral neuropathy demonstrated the highest significant OR (95% CI) at 8.03 (5.47–11.78) followed by insulin usage, age ≥45 years, diabetes duration ≥10 years, retinopathy, and poor glycemic control. Charcot joint, PVD, and nephropathy had high, but non-significant OR, whilst CVD, CAD, and hypertension did not demonstrate any significant risk for diabetic foot complications, see Table 4.

**Table 4:** Age and gender—adjusted and multivariate—adjusted odds ratio and 95% confidence intervals of risk factors in the studied cohort.

Risk factors	Age and gender adjusted			Multivariate adjusted		
	OR	95% CI	p-value	OR	95% CI	p-value
Age ≥ 45 yrs	-	-	-	3.81	2.22–6.54	<0.0001
Male gender	-	-	-	1.92	1.49–2.48	<0.0001
Charcot joint	38.80	16.20–92.93	<0.0001	3.17	0.38–26.42	0.285
Peripheral vascular disease	11.60	7.16–18.79	<0.0001	2.69	0.29–24.57	0.380
Peripheral neuropathy	9.71	8.43–11.16	<0.0001	7.20	4.84–10.71	<0.0001
DM Duration ≥10 yrs	6.69	5.64–7.93	<0.0001	2.50	1.66–3.77	<0.0001
Insulin use	4.99	4.56–5.48	<0.0001	3.98	3.02–5.23	<0.0001
Retinopathy	3.88	3.53–4.27	<0.0001	1.84	1.43–2.35	<0.0001
Nephropathy	3.66	3.31–4.04	<0.0001	1.26	0.94–1.69	0.129
Poor glycemic control	2.65	2.08–3.39	<0.0001	1.49	1.12–1.98	0.006
Cerebral vascular disease	2.30	1.89–2.81	<0.0001	0.63	0.28–1.39	0.251
Coronary heart disease	1.86	1.64–2.11	<0.0001	0.92	0.63–1.36	0.688
Hypertension	1.35	1.24–1.48	<0.0001	1.04	0.81–1.33	0.779
Smoking	1.15	0.96–1.37	0.120	-	-	-

## Discussion

This retrospective registry-based study shows the prevalence of diabetic foot complications among the diabetic patients cohort to be at 3.3%, out of which 2.05% were diabetic foot ulcer cases, which is within the estimated international range (1.8% to 7%) [18,19]. Amputation in this cohort was at a rate of 1.06% which is also similar to the findings reported elsewhere (0.9% in Slovakia and 3% in Canada) [20,21]. This study reported the prevalence of foot gangrene at a much lower rate than what has been reported by the Rochester, MN study, at 0.8% in USA [22] or by Rabia et al., at 3% in Malaysia [23]. This could be explained by the fact that the diabetic population in this study was selected from different hospital departments including primary care clinics compared to only diabetes or foot clinics in the other studies.

### Age and gender effect

The prevalence of all diabetic foot complications increased clearly with age and diabetes duration, regardless of its types, as observed by others [4, 24–26]. The mean age played an important role in the occurrence of foot ulcer or gangrene, wherein 50% of the cases were older than 65 years. This was also the observation from other studies, where the prevalence of diabetic foot ulcer varied between 1.7 to 3.3% in younger patients and 5 to 10% among older patients [27]. Amputation rate also increased with age similar to what has been reported by Katsilambros et al., where it was 1.6% in the age 18–44 years, 3.4% in the age 45–64 years, and 3.6% in patients older than 65 years [4]. The vast majority of diabetic foot cases in the current analysis had diabetes duration more than 10 years similar to Moss et al. findings [26], which also holds true for foot ulcer, gangrene, and amputation cases.

The total and age-specific prevalence of foot ulcer, gangrene and amputation was significantly higher in males than females as shown in many studies [28,29] and could by explained on the basis that, males are known to have limited joint mobility and higher foot pressure. Higher mean height and peripheral insensate neuropathy found more frequently in males could contribute to this difference [30,31]. In contrast, women are more self-caring and have a positive mood in terms of being active with body care, while males express fear and negative attitudes [32]. This is in addition to the fact that, males are more exposed to trauma and tend to wear improper footwear, especially in our culture [33,34]. However, this was not the case with the age-specific gangrene prevalence, where there was no significant difference between the two genders, which could be the effect of the small number of cases in each age group.

### Diabetes type, duration, and control effect

As expected, and reported by others, the percentage of type 2 diabetic patients was more among diabetic foot cases in the current study [35,36], while the prevalence of diabetic foot was higher among type 1 diabetic patients at 4.53% versus 3.55% in type 2 diabetic patients. This could be explained by longer diabetes duration and higher rate of chronic complications, especially neuropathy among type 1 diabetic patients [37]. There has been a clear and significant relation between the three diabetic foot conditions and the degree of glycemic control, which is in consistence with the observation that, poor glycemic control was associated with two-fold increase in the risk of foot lesions among diabetic patients [38]. In terms of diabetes management, the foot cases were frequent users of insulin, which is consistent with other studies [39,40], and as expected, since poorly controlled foot cases will require insulin treatment.

### Chronic complications

The frequency of chronic complications was significantly higher among our diabetic foot cases, especially neuropathy which affects 61.98% of foot ulcer cases as reported by Grunfeld [41]. Cases with CAD showed the highest percentage among vasculpoathic patients for foot ulcer, amputation and gangrene. This finding could be explained on the basis of high prevalence of CAD among our diabetic population contributing to 23.56% of affected patients, in addition to the fact that CAD is known to be highly prevalent among diabetic foot cases [42]. However, PVD contributed to one third of foot ulcer cases in the studied cohort, which is similar to what has been previously reported [43] and was responsible for more than 50% of the amputation cases.

Diabetic retinopathy and nephropathy increased the percentage of the three foot complications in the current study, that could be explained by microangiopathic changes [44], which is also the same observation from other studies [26,29]. Renal impairment could contribute to foot lesion and/or delay healing process [45], while decreased vision associated with retinopathy might increase the chance of foot trauma [46].

In line with other observations, our study showed that more than 50% of ulcer, gangrene, and amputation cases occurred in hypertensive patients [39,40]. On contrary, hyperlipidemia was found to be less prevalent in the total cases affected with foot ulcer, gangrene and amputation when compared with the non-affected cases. This could be explained by the fact that, majority of cases were controlled with lipid lowering agents during the time of analysis.

The current analysis found smoking to be associated with foot ulcer and gangrene which was also observed from several studies [47,48], whilst it was not the case for amputees. Although this was the same findings of Akha et al among Iranian population [49], this observation contradicts with many other studies that reported significantly higher frequency of smokers among amputees [50,51]. This could be explained by the fact that, all these patients were identified as foot ulcer or gangrene cases before they went for amputation which may have altered their smoking habits [52]. This is in addition to the fact that, smoking among females is socially unacceptable in our society which would evidently decrease the percentage of smokers in the total cohort [53].

The mean BMI was significantly lower among diabetic foot ulcer, gangrene, and amputation cases, which was also observed with the Americans and Costa Ricans [35,39]. This could be explained by the fact that, weight loss is associated with chronic complications like nephropathy which is observed in about one third of all affected cases, in addition to the fact that, these affected cases are having a significantly higher mean height that would lower their BMI value [54].

### Risk factors

The presence of Charcot joint significantly increased the risk for foot ulcer and amputation, but not gangrene in the univariate model. This was consistent with the findings of Sohn et al., that showed seven folds increase in the relative risk for amputation among Charcot joint patients [54]. Boyako et al., on the other hand, showed almost 4-folds increase in foot ulceration risk with patients suffering from Charcot joints [55]. Charcot disease is not associated with any risk for foot gangrene since it is a neuropathic disorder as shown by our study. However when adjusting for other risk factors using multivariate analysis this strong association has reduced with a non- significant association. As reported in many other studies [29,40], our study also showed that PVD was associated with significantly increased risk of all types of diabetic foot complications. This was true for univariate, age and gender adjusted, and multivariate models, but this association was not found to be significant in the multivariate analysis, as previously reported in our society by Abolfotouh et al. [40]. Peripheral neuropathy is one of the strongest risk factors for all the foot complications amongst the studied cohort, with this association also being significant in age and gender adjusted and multivariate logistic regression models as having found in Danish and Saudi populations [29,40]. This strong association of the PVD and peripheral neuropathy with diabetic foot complications could reflect the high prevalence of peripheral nerve decompression among Saudi diabetic patients, especially when it has been reported elsewhere that, 33% of diabetic patients are suffering from chronic nerve compression [56]. However, this observation could shed light on the importance of considering screening for lower extremities nerve compression among diabetic patients and applying the recently addressed concept of surgical nerve decompression at lower extremity (neurolysis of tibial nerve and its branches in tarsal tunnel) that has been proven to significantly prevent new ulcers and amputations through improving nerve function and increasing microcirculation [57,58], which can be evaluated and followed up by transcutaneous oximetry that overpassed the limit defined of tissue hypoxia [59].

Other chronic diabetes complications, namely nephropathy, retinopathy, CVD and CAD were significantly associated with increased risk of diabetic foot ulcer, gangrene, and amputation. This positive association was also observed amongst Danish, Mexican, and Turkish populations [29,35,36]. However, this association remained significant only with diabetic retinopathy in the multivariate model, which was also the same observation amongst the Danish population [29]. The positive and strong association between diabetes duration and risk of foot complications as seen in our study is consistent with the findings of Moss et al. and Lavery et al. [26,35] wherein, diabetes duration of ≥10 years significantly increased the risk for foot ulceration and amputation by 3 to 4 folds.

Pursuant with the findings of other researchers [40,35], age ≥45 years and male gender were significant non-modifiable risk factors that showed increased risk for all types of foot complications in both univariate and multivariate logistic regression models. Insulin use and poor glycemic control increased the risk of diabetic foot complications in our study by almost five-folds and three-folds in univariate analysis, and this significant risk remained in the multivariate adjusted model similar to the Seattle diabetic foot study and Lavery et al., findings [55,35]. Hypertension and smoking in our study, significantly increased the risk for foot ulcer and gangrene as similar to the observational findings amongst the Taiwanese and Turkish populations [47,36]. On the other hand, smoking was not a significant risk factor for amputation for the reason explained earlier, which is consistent with the findings of a prospective study conducted in Costa-Rica [39]. In the univariate analysis, obesity was associated with significantly reduced risk for all diabetic foot complications. This finding is supported by the phenomenon called " obesity paradox" observed by Ledoux et al., wherein, 5 Kg/m^2^ increment in BMI was associated with reduced risk for foot ulcer [60], and also supported by the findings of Sohn et al., where they reported the lowest risk among overweight and class I obesity (BMI 25–34.9 kg/m^2^) [61]. Additionally, this observation was found among amputees contradicting with what other researchers have reported [62], but consistent with the most recent findings of Sohn et al. in a large male cohort [63], and can be explained by Biasucci et al findings that obese people may have better wound healing [64].

Our study is limited by its hospital-based retrospective nature that lacks certain specific information and being a cross-sectional study which is not the right set up for determining causality. Despite these limitations, our study is derived from a large web-based electronic registry focusing on diabetes and its complications with frequent follow ups and data validation. This large cohort provided enough number of cases for better analysis.

## Conclusions

In conclusion, the prevalence of diabetic foot complications in Saudi population from this large database sets within what is reported internationally. Diabetic foot ulcer cases contributed to more than 50% of the total diabetic foot cases. The presence of peripheral neuropathy and PVD is considered to be the most significant risk factors for all types of diabetic foot complications. This study has confirmed the importance of previously known risk factors for diabetic foot complications, in addition to demonstrating the importance of diabetic retinopathy as a significant independent risk factor that has to be taken into account during screening for foot problems in diabetic patients. Since those risk factors are highly prevalent in our diabetic population, primary and secondary prevention programs are urgently needed to minimize both morbidity and cost from this chronic complication. In addition to controlling risk factors, other measurements like decompression of lower extremity nerves should be considered among diabetic patients who are most likely suffering from unrecognized lower extremity chronic nerve compression that would have a positive effect on improving nerve function and microcirculation.
